# Cytotoxic Activity of *Halymenia durvillaei* against Cervical Cancer Cells: In Vitro and Computational Evidence

**DOI:** 10.1007/s12013-026-02099-9

**Published:** 2026-06-17

**Authors:** Grace Sanger, Lena J. Damongilala, Vincent Lau, Raffaele Romano, Antonello Santini, Fahrul Nurkolis

**Affiliations:** 1 Department of Fishery Product Technology, Faculty of Fishery and Marine Science, Sam Ratulangi University, Manado, North Sulawesi, 95115 Indonesia; 2 Faculty of Medicine, Public Health, and Nursing, Universitas Gadjah Mada, Yogyakarta, 55281 Indonesia; 3 Department of Agricultural Sciences, University of Napoli Federico II, Piazza Carlo di Borbone 1, 80, Portici, 055 Italy; 4 Department of Pharmacy, University of Napoli Federico II, Via Domenico Montesano, 49, Napoli, 80131 Italy; 5 Faculty of Medicine, Universitas Airlangga, Surabaya, Indonesia; 6 State Islamic University of Sunan Kalijaga (UIN Sunan Kalijaga), Yogyakarta, 55281 Indonesia; 7 Medical Research Center of Indonesia, Surabaya, Indonesia

**Keywords:** *Halymenia durvillaei*, Red seaweed, Marine natural products, Cytotoxicity, Cervical cancer, HeLa cells, Brine shrimp lethality test, GC–MS profiling, Mmolecular docking, VHR phosphatase

## Abstract

Cervical cancer remains a major global health burden, particularly in low- and middle-income countries, necessitating the discovery of novel, accessible anticancer agents. Marine macroalgae, including *Halymenia durvillaei*, are rich in bioactive compounds with promising therapeutic potential. H. durvillaei collected from North Sulawesi, Indonesia, was extracted with methanol and fractionated into n-hexane, chloroform, ethyl acetate, and aqueous fractions. Phytochemical profiling and cytotoxicity were assessed using the brine shrimp lethality test (15.625–1000 µg/mL) and MTT assay against HeLa cells treated with extract fractions (0.125–2 mg/mL) for 72 h. The most bioactive fraction was subjected to GC–MS characterization. Major compounds identified were evaluated through molecular docking against Vaccinia H1-related phosphatase (VHR; PDB ID: 3F81) using AutoDock Vina/CB-Dock2, followed by 100 ns molecular dynamics simulation and ADMET prediction. Phytochemical screening confirmed the presence of flavonoids (2.91 mg QE/g), alkaloids (1.03 mg/g), tannins, saponins, and steroids. The chloroform fraction exhibited the highest cytotoxicity (LC₅₀ = 33.83 µg/mL), while ethyl acetate, n-hexane, chloroform, and aqueous fractions inhibited 70–93% of HeLa cell proliferation. GC–MS analysis identified oleic acid as a major compound. In silico studies revealed stable binding of oleic acid to VHR phosphatase, a protein implicated in cervical cancer progression, supported by favorable ADMET properties. H. durvillaei demonstrates significant cytotoxic and antiproliferative activities, highlighting its potential as a source of natural anticancer agents. However, further compound isolation and mechanistic validation are required to confirm its therapeutic relevance.

## Introduction

Cervical cancer remains one of the leading causes of cancer-related deaths among women, particularly in low- and middle-income countries, where access to screening and treatment is limited [1, 2]. Cervical cancer is the most common cancer among women, representing 12% of the total cancer burden and the leading cause of cancer-related deaths [1]. Globally, cervical cancer ranks as the fourth most common cancer among women, with approximately 660,000 new cases and 350,000 deaths reported annually [3]. In Southeast Asia, cervical cancer remains a major public health concern, with substantial incidence and mortality rates that continue to burden healthcare systems, particularly in low- and middle-income settings [4]. Although the burden has generally declined, cervical cancer remains a major public health concern, with the age-standardized incidence rate (ASIR) decreasing from 180.0 (95% CI: 139.0–226.0) per 100,000 in 1990 to 131.0 (95% CI: 96.6–170.0) in 2021 [5]. In Indonesia, cervical cancer continues to represent one of the most common cancers among women, highlighting the need for accessible preventive and therapeutic strategies [6]. The persistent burden of this disease highlights the urgent need for novel, accessible, and natural therapeutic agents.

Marine macroalgae have emerged as promising sources of bioactive compounds with anticancer potential due to their structural diversity and bioactivity, including antioxidant, anti-inflammatory, and antiproliferative effects. In recent years, the research and demand for seaweed products as anticancer agents has escalated. Various mechanisms for the modulation of carcinogenesis by seaweed have been suggested [7–9]. Sulfated polysaccharides, notably fucoidan and alginates, are extensively researched components in seaweeds for their antitumor properties. Polysaccharides can obstruct cell cycle progression, trigger apoptosis, and impede metastasis in tumor cells [10]. The capacity of these polysaccharides to stimulate immune responses and regulate signaling pathways emphasizes their therapeutic potential [11]. Besides polysaccharides, terpenoids present in seaweeds have been recognized as potential antitumor agents. Chen’s literature review highlights the function of terpenoids in suppressing malignant tumor traits, including unchecked cell proliferation and invasion [12]. This corresponds with findings that specific terpenoids can induce apoptosis and augment the efficacy of current cancer therapies [13, 14].

*Halymenia durvillaei* (*H. durvillaei*), a species of red algae, has gained considerable attention in recent years due to its rich content of bioactive compounds and potential therapeutic applications, particularly in the field of oncology. This macroalga is predominantly found in the coastal regions of the Indo-Pacific, where it has adapted to challenging environmental conditions, resulting in the synthesis of diverse secondary metabolites with significant biological activities [15, 16]. The bioactive compounds extracted from *H. durvillaei* include phenolic compounds, polysaccharides, and alkaloids, which have demonstrated antioxidant, anti-inflammatory, and anticancer properties [17, 18]. The unique chemical composition of *H. durvillaei*, particularly its high levels of sulfated polysaccharides and phenolic compounds, enhances its potential as a functional ingredient in nutraceutical applications targeting cancer prevention and treatment [17, 19]. The investigation of *H. durvillaei* in the context of human cervical adenocarcinoma aligns with a growing body of evidence supporting the use of marine algae as a source of novel anticancer agents [20, 21].

Despite the growing interest in the pharmacological potential of marine macroalgae, studies specifically addressing the cytotoxic and anticancer activities of *H. durvillaei* remain limited, particularly concerning its effects against human cervical adenocarcinoma (HeLa) cells. Previous studies on red algae have primarily focused on general anticancer, antioxidant, or anti-inflammatory properties, while investigations of *H. durvillaei* have largely emphasized nutritional composition, broad bioactivity, or effects on other cancer models. Moreover, integrated evaluations combining preliminary toxicity screening, cancer cell cytotoxicity, phytochemical characterization, and computational target interaction analysis in *H. durvillaei* are still lacking. To address this gap, the present study aims to evaluate the cytotoxicity, phytochemical profile, and in-silico molecular interactions of *H. durvillaei* extracts and fractions, with a focus on identifying compounds responsible for antiproliferative activity. The novelty of this research lies in its integrated approach, combining in-vitro assays (Brine Shrimp Lethality and MTT cytotoxicity tests) with in-silico molecular docking and ADMET analyses, to elucidate the potential mechanism of action of bioactive compounds derived from *H. durvillaei*. This study provides new insights into the potential use of red algae-derived polysaccharides and secondary metabolites as alternative or complementary therapeutic agents for cervical cancer treatment.

## Materials and Methods

### Sample Collection and Preparation

The seaweed *H. durvillaei* was harvested from the coastline waters of North Sulawesi, Indonesia (coordinates: 01°09′22″ N, 125°01′32″ E) in January 2026 under tropical coastal conditions during low tide. The collected specimen was taxonomically identified and authenticated by Prof. Dr. Grevo S. Gerung, M.Sc. (Faculty of Fisheries and Marine Science, Sam Ratulangi University, Manado, Indonesia), based on morphological characteristics and taxonomic references. A voucher specimen (Voucher Number HD-2026-01) has been deposited at the Herbarium of Sam Ratulangi University for future reference. The preparation and extraction of the samples were conducted according to the methodologies outlined by Sanger et al., [22] and Gomez et al., (2015) [22, 23]. Upon collection, the samples were promptly cleaned and washed with seawater to remove any sand, debris, epiphytes, or other extraneous substances that may have adhered to the thalli. Thereafter, the purified samples were transferred to the laboratory for the analysis. The samples were washed with distilled water, and sectioned into small fragments. All samples were prepared in triplicate. A total of 500 g of each sample were extracted in 1 L of methanol (Merck, Darmstadt, Germany) and agitated in the dark for 24 h. The extracts were filtered and then subjected to rotary evaporation (Büchi Rotavapor R-300, Flawil, Switzerland) at 40 °C until concentrated extracts were obtained. The methanolic extract (ME) of *H. durvillaei* was subsequently fractionated successively with n-hexane, chloroform, ethyl acetate, and distilled water using a separatory funnel (Pyrex^®^, Corning Inc., Corning, NY, USA). Each fraction was filtered and concentrated using the same rotary evaporator at 40 °C.

Five hundred g of each seaweed sample were rinsed, divided into small pieces, and suspended in 1 L of ethanol. Then the sample was then shaked for 24 h in the dark using an orbital shaker (IKA KS 4000, IKA-Werke GmbH & Co. KG, Staufen, Germany). The solvent extracts were filtered using Whatman No. 1 filter paper (Cytiva, Marlborough, MA, USA) and concentrated under reduced pressure using a rotary evaporator (Büchi Rotavapor R-300, Büchi Labortechnik AG, Flawil, Switzerland) at 40 °C. The crude methanolic extraction yielded 63.5 g extract (12.7%, w/w) from 500 g dried seaweed biomass. Subsequent liquid–liquid partitioning generated n-hexane, chloroform, ethyl acetate, and aqueous fractions with yields of 5.8 g (1.16%), 8.9 g (1.78%), 11.6 g (2.32%), and 31.2 g (6.24%), respectively.

Fractionation was performed sequentially based on solvent polarity using liquid–liquid partitioning of the crude methanolic extract, beginning with n-hexane (non-polar), followed by chloroform and ethyl acetate (semi-polar), and finally the aqueous fraction (polar) to separate compounds according to their polarity characteristics. The resultant methanol extracts (ME) were then dissolved in dimethyl sulfoxide (DMSO) to ensure complete solubilization and subsequently diluted with Dulbecco’s Modified Eagle Medium (DMEM) to the desired concentrations prior to cell treatment. DMSO was used as a solvent carrier at a final concentration of ≤ 0.1% (v/v) in all treatment groups to minimize solvent-induced cytotoxicity, while vehicle controls received an equivalent DMSO concentration. Triplicate measurements referred to independent analytical and biological assay replicates rather than repeated extraction and fractionation processes. The crude extraction and solvent partitioning were performed once using a representative bulk sample, while all subsequent experimental assays were conducted in triplicate.

### Phytochemical Quantitative Analysis

#### Alkaloid Content

The alkaloid content in the extract was quantified using a previously established method [24]. Ten grams of the extract were dissolved in 100 mL of methanol containing 2% acetic acid (Merck, Darmstadt, Germany). Five milliliters of the test solution were mixed with two milliliters of Dragendorff’s solution (Sigma-Aldrich, St. Louis, MO, USA) from the prepared mixture. The resulting mixture was centrifuged, and the precipitate was washed with 5 mL of ethanol. Subsequently, 2 mL of disodium sulfide were added, followed by further centrifugation. The samples were centrifuged at 5,000 rpm (approximately 3,000 × *g*) for 10 min at 4 °C, and the resulting supernatant was collected for subsequent analysis. The supernatant was discarded, and the precipitate was washed again with 5 mL of ethanol. The precipitate was solubilized in 2 mL of nitric acid and subsequently diluted with distilled water to achieve a final volume of 10 mL. The extract or standard was combined with 5 mL of a 3% thiourea solution. A calibration curve was generated using a standard solution of bismuth nitrate Bi(NO_3_)_3_ ·5 H_2_O with concentrations ranging from 2.5 to 20 µg/mL. Bismuth nitrate was selected as the calibration standard because the alkaloid assay employed was based on a bismuth-complexation/colorimetric reaction commonly used for estimating total alkaloid content in plant and marine extracts. Accordingly, the obtained values should be interpreted as relative total alkaloid equivalents rather than absolute quantification of individual alkaloid compounds. The absorbance of the mixture was quantified at a wavelength of 435 nm using a UV-Vis spectrophotometer (Shimadzu UV-1800, Kyoto, Japan), and the alkaloid concentration was expressed as mg/g of extract.

#### Flavonoid Content

The total flavonoid content (TFC) of each extract was determined using the aluminum chloride colorimetric method, which is based on the formation of stable complexes between flavonoids and aluminum ions, resulting in a measurable yellow coloration [25]. The extract was dissolved in methanol to obtain a concentration of 100 µg/mL. Calibration curves were prepared using quercetin dissolved in methanol at concentrations ranging from 0 to 100 µg/mL. Subsequently, 2 mL of the extract solution or quercetin standard were mixed with 0.1 mL of 10% aluminum chloride solution and 0.1 mM potassium acetate solution. A 0.1 M potassium acetate solution was prepared by dissolving potassium acetate in distilled water and used as a reagent in the aluminum chloride colorimetric assay for flavonoid quantification. The mixture was allowed to stand at room temperature for 30 min, and absorbance was measured at 415 nm. The total flavonoid content was expressed as milligrams of quercetin equivalents per gram of extract (mg QCE/g extract).

#### Tannin Content

The Folin-Denis method was employed to quantify the tannin content [26]. This colorimetric method entails quantifying the blue hue generated from the reduction of phosphomolybdic-phosphotungstic acid by tannins in an alkaline environment. A standard solution of tannic acid (100–800 µg/mL) or an extract was incorporated into 7.5 mL of distilled water. A 7.5% (w/v) sodium carbonate (Na₂CO₃) solution was prepared by dissolving 7.5 g Na₂CO₃ in 100 mL distilled water and used in the Folin–Denis assay to facilitate color development during tannin quantification. After incorporating 0.5 mL of Folin-Denis reagent and 1 mL of Na_2_CO_3_ solution, the total volume was adjusted to 10 mL with distilled water. The absorbance was recorded at a wavelength of 750 nm. The total tannin content was quantified as tannic acid equivalent per gram of dry weight.

#### Saponin Content

The saponin content was quantified using the Liebermann-Burchard colorimetric method, which is based on the reaction between saponins and acetic anhydride-sulfuric acid, producing a green-colored complex measurable by spectrophotometry, as previously described [27, 28]. One hundred mg portion of each extract was first extracted with diethyl ether (Merck, Darmstadt, Germany) to remove nonpolar impurities. The ether extract was then evaporated to dryness at 40 °C for 24 h in a hot-air oven (Memmert UF55, Schwabach, Germany) until all solvent residues were removed and a consistent dry mass was obtained. The dried residue was redissolved in 10 mL of ethanol (analytical grade, Merck, Germany). The saponin concentration was ascertained by combining 1 mL of extract or standard with 3.5 mL of Lieberman–Burchard reagent. The solution was vortexed, stored in a dark room at ambient temperature for 30 min, and subsequently, the wavelength was measured at 435 nm. Total saponin content was quantified using diosgenin as the calibration standard, and the results were expressed as milligrams of diosgenin equivalents per gram of extract (mg DE/g extract).

#### Steroid Content

The Liebermann-Burchard test is employed to ascertain steroid content, as previously described [29]. The Liebermann–Burchard assay employed concentrated sulfuric acid (H₂SO₄, 95–98%, analytical grade) as a reagent for color development during steroid quantification. In particular, a cholesterol solution at concentrations of 5, 10, 15, 20, 25, and 30 µg/mL was utilized as a control. After incorporating 2 mL of anhydrous acetic acid and 0.1 mL of H_2_SO_4_, the solution was homogenized using a vortex mixer. The solution was subsequently covered with aluminum foil to shield it from light and allowed to stand for 15 min. To ascertain the total steroid content of the extract, 10 mg of the sample was weighted and a 96% ethanol was added to a 10 mL volumetric flask until the calibration mark was reached. Subsequently, 0.15 mL of each sample was transferred into a 5 mL volumetric flask. The flask was subsequently filled to the mark with 96% ethanol following the addition of 2 mL of acetic anhydride. The absorbance was quantified at a wavelength of 423 nm. The concentrations of steroids were quantified as milligrams of cholesterol equivalent per gram of extract.

### Brine Shrimp Lethality Test

A Brine Shrimp Lethality Test (BSL) was performed to assess the cytotoxicity of the extract and fraction of *H. durvillaei* against nauplii of *Artemia salina* (Sanders Great Salt Lake, Brine Shrimp Company, L.C., USA), following a previously established methodology with minor modifications [30, 31]. Artificial seawater used for hatching and maintaining Artemia salina nauplii was prepared by dissolving commercial marine salt in distilled water to achieve a salinity of 30–35 ppt, simulating natural seawater conditions suitable for nauplii survival during the assay. Dried cysts were incubated in artificial seawater, and after approximately 12 h, phototropic nauplii were harvested and concentrated in small vials. Each test group consisted of 15 nauplii exposed to different concentrations of methanol, n-hexane, chloroform, and aqueous extracts at 15.625, 31.25, 62.5, 125, 250, 500, and 1000 µg/mL. Water functioned as a negative control. Although methanol and organic solvents were used during extraction and fractionation, all solvents were completely removed under reduced pressure prior to biological testing. Before the assay, extracts were reconstituted in DMSO and diluted to working concentrations, with the final DMSO concentration maintained at ≤ 0.1% (v/v). Accordingly, a solvent-matched vehicle control containing an equivalent DMSO concentration was included alongside the water control to exclude potential solvent-induced toxicity. The toxicity of the extracts was assessed by enumerating the surviving nauplii and calculating the mortality percentage after 48 h. Nauplii were deemed deceased if they remained immobile for 30 s. Bioassays were conducted with three replicates. Mortality percentages and LC_50_ values were determined through probit analysis and concentration versus lethality percentage using the Finney computer program BioStatTM 2009 (Analysis Soft Inc., Vancouver, Canada).

### Cytotoxicity Activity

The cytotoxic efficacy of the extract was assessed using the MTT assay [23]. The MTT assay relies on the reduction of MTT by the mitochondrial dehydrogenases of viable cells, resulting in the formation of a purple formazan compound. HeLa cervical adenocarcinoma cells (ATCC^®^ CCL-2™) were obtained from the American Type Culture Collection (ATCC, Manassas, VA, USA) and cultured in Dulbecco’s Modified Eagle Medium (DMEM) supplemented with 10% fetal bovine serum (FBS), 100 U/mL penicillin, and 100 µg/mL streptomycin under standard culture conditions. Ethical approval was not required because the experiments were performed using a commercially available immortalized human cell line (ATCC^®^ CCL-2™). Cells were sustained as monolayer cultures in a humidified environment with 5% CO_2_ at 37 °C. HeLa cells were seeded at a density of 5 × 10⁵ cells in 75 cm² culture flasks containing DMEM supplemented with fetal bovine serum and incubated under standard culture conditions. HeLa cells were collected and inoculated into 96-well plates at a density of 5 × 10^*3*^ cells per well in 100 µL of serum-free medium, incubated at 37 °C with 5% CO_2_ for 24 h. To ensure experimental reliability, HeLa cells were maintained under aseptic conditions and regularly monitored for contamination during culture. Experiments were conducted using cells at passages 5–15, and the cell line was obtained from an authenticated commercial/institutional source. The medium was subsequently removed, and 100 µL of test solution (ME) at varying concentrations, along with 100 µL of medium containing 5% FCS (MM), was introduced to the plate. Cells were cultured for 72 h at 37 °C with a 5% CO_2_ concentration. Cells solely encased by MM served as the control group. Following incubation, surplus ME was removed by washing the cells twice with 200 µL of PBS and employing MTT (5 mg/mL) dissolved in 100 µL of fresh medium to assess the impact of the ME on cell viability. *H. durvillaei* extracts and fractions were tested at concentrations of 15.625, 31.25, 62.5, 125, 250, 500, and 1000 µg/mL. The cells were subsequently incubated for 4 h at 37 °C in a 5% CO_2_ environment. Following treatment, MTT solution was added to each well and incubated for 3–4 h at 37 °C to allow intracellular reduction of MTT into formazan crystals. The culture medium was then carefully removed, and the resulting formazan product was dissolved in DMSO prior to absorbance measurement at 570 nm using a microplate reader (Multiskan™ Ascent, Thermo Fisher Scientific, Waltham, MA, USA). Microscopic morphological evaluation of HeLa cells was not systematically performed in the present study; therefore, cytotoxicity assessment was based primarily on the MTT assay.

Cell viability was calculated using the following equation:

$$\:Cell\:Viability\left(\%\right)=\frac{Absorbance\:of\:treate\:cells}{Absorbance\:of\:control\:cells}x100$$

Cell growth inhibition (%) was determined as:

$$\:Inhibition\left(\%\right)=100-Cell\:Viability\left(100\%\right)$$

The half-maximal inhibitory concentration (IC₅₀) was estimated from dose–response curves by plotting extract concentration against percentage inhibition using nonlinear regression analysis.

### Bioactive Compounds of H. durvillaei using GC–MS-Based Chemical Characterization of the Active Fraction

To chemically characterize the fraction associated with the strongest bioactivity, GC–MS analysis was performed on the most active fraction of *H. durvillaei* [32] using an Agilent 7890B gas chromatograph coupled with an Agilent 5977 A mass selective detector (Agilent Technologies, Santa Clara, CA, USA). The dried fraction was dissolved in HPLC/GC-grade n-hexane, vortex-mixed, and filtered through a 0.22 μm PTFE membrane prior to injection. The analysis was conducted using a GC–MS system operating in electron impact ionization mode (70 eV) and equipped with a non-polar fused-silica capillary column (30 m × 0.25 mm, 0.25 μm). Helium was employed as the carrier gas at a constant flow rate of 1.0 mL/min. The injector temperature was set at 250 °C and the injection volume was 1 µL. The oven temperature was programmed from 60 °C to 300 °C at 10 °C/min and held at the final temperature to ensure elution of late-retained constituents.

Mass spectra were acquired over an m/z range of 40–600. Tentative identification of volatile and semi-volatile compounds was achieved by matching the acquired spectra against the NIST mass spectral database and by comparing retention behavior and fragmentation patterns with previously reported data. The composition of the fraction was expressed as relative peak area percentages from the total ion chromatogram. Only compounds consistently detected and showing satisfactory library similarity scores were included in the list of putatively identified constituents. Compounds detected as major peaks were selected for subsequent computational analysis.

The computational findings were used as a preliminary approach to assess the potential interaction between the identified compound and the selected molecular target. Oleic acid was included as a major bioactive constituent due to its recurrent identification as a dominant fatty acid in *H. durvillaei* extracts, particularly in lipid-rich fractions analyzed by GC–MS and FAME profiling [33]. Oleic acid was selected because it represented one of the predominant compounds identified by GC–MS and has been previously reported to exhibit biological activities, including antiproliferative and anticancer-related effects. The selection of oleic acid for further analysis was supported by both its abundance in *H. durvillaei* and its well-documented biological relevance in metabolic and inflammatory pathways.

### Molecular Docking

To provide a preliminary and hypothesis-generating assessment of possible molecular interactions, compounds identified as major constituents in the GC–MS profile of the active fraction were subjected to molecular docking analysis. Docking was performed only for compounds experimentally detected in the analyzed fraction.

The three-dimensional structures of the ligands were retrieved from PubChem in SDF format and prepared prior to docking. The crystal structure of vaccinia H1-related phosphatase (VHR) was obtained from the Protein Data Bank (PDB ID: 3F81). VHR, a dual-specificity phosphatase involved in cell cycle regulation and MAPK signaling, has been implicated in cervical cancer progression and cell proliferation. Molecular docking was performed using the CB-Dock2 platform, which automatically identifies potential binding cavities and generates grid box dimensions based on the predicted active pocket of the target protein. Docking calculations were conducted using the AutoDock Vina engine with an exhaustiveness value of 8, and up to 10 binding modes were generated for each ligand to evaluate potential binding conformations. The molecular docking analysis was conducted using the CB-Dock2 server, which is based on AutoDock Vina version 1.1. at the following URL: https://cadd.labshare.cn/cb-dock2/php/blinddock.php [34–36].

Before docking, the VHR protein structure obtained from the Protein Data Bank (PDB ID: 3F81) was prepared by removing water molecules and unnecessary heteroatoms, while hydrogen atoms were added to facilitate docking interactions. Protein preprocessing and structural optimization were performed within the CB-Dock2/AutoDock Vina workflow prior to docking analysis. Blind docking was applied to detect potential binding cavities and estimate the binding affinity of each ligand toward the target protein. The co-crystallized ligand was re-docked to evaluate the reliability of the docking protocol, and the root mean square deviation (RMSD) between the native and re-docked poses was calculated. The ligands utilized in the study were previously documented, and oleic acid was incorporated into this molecular docking analysis and ADMET assessment (https://preadmet.qsarhub.com/) [32]. Oleic acid was selected for docking and ADMET analysis because it represented one of the predominant compounds identified by GC–MS and served as a representative candidate for preliminary computational assessment. However, the cytotoxic activity of H. durvillaei is likely influenced by multiple constituents, and additional compounds warrant further investigation in future studies. Three-dimensional (3D) SDF files of the compounds were obtained from PubChem. The protein and compounds were submitted to the CB-Dock2 server, and a cavity detection-based blind docking was performed [37]. Docking interaction figures and protein–ligand interaction analyses were generated using Discovery Studio Visualizer v21.1.0.20298 (BIOVIA, San Diego, CA, USA).

It should be noted that this docking analysis was used only as an exploratory computational approach to predict possible ligand–protein interactions and did not serve as experimental confirmation of the molecular target. Therefore, the docking results were interpreted cautiously and only in conjunction with the in vitro cytotoxicity data.

### Molecular Dynamics Simulations

A 100-nanosecond molecular dynamics (MD) simulation was performed utilizing the Gromacs-based WEBGRO server (https://simlab.uams.edu/index.php) to examine the stability of the chosen compound, VHR complex, and apoprotein [38, 39]. WEBGRO was employed as a user-friendly GROMACS-based platform for preliminary molecular dynamics analysis of protein–ligand stability under standardized conditions. While it provides less parameter flexibility than locally executed GROMACS simulations, it enables reproducible assessment of key stability indicators, including RMSD, RMSF, and hydrogen bond interactions. The chosen compound was derived from molecular docking results, exhibiting the greatest affinity for the receptor. Molecular dynamics trajectory stability was evaluated using RMSD, RMSF, radius of gyration (Rg), hydrogen bond analysis, and solvent-accessible surface area (SASA). These parameters were analyzed to assess structural stability, residue flexibility, intermolecular interactions, and compactness of the protein–ligand complex during simulation. The VHR protein topology was constructed utilizing the GROMOS 54A7 force fields, whereas the oleic acid topology file was produced via the GlycoBioChem PRODRG2 Server (http://davapc1.bioch.dundee.ac.uk/cgi-bin/prodrg/) [40, 41]). A triclinic box was created at a distance of 10 Å from the protein-ligand complex, utilizing SPC water for solvation, and it was neutralized by the addition of 0.15 M NaCl. Energy minimization was executed utilizing the steepest descent integrator for 5000 iterations. Equilibration was attained at a temperature of 27 °C and a pressure of 1 bar utilizing the NVT/NPT ensemble for a duration of 0.3 ns. A total of 1000 frames of 100 ns molecular dynamics simulation were conducted utilizing the Leap-frog MD integrator. The MD plots were visualized with QtGrace tool v0.2.6, and the animation videos were generated using UCSF Chimera v1.16 [42].

### Statistical Analysis

All experimental measurements were performed in technical triplicate, referring to repeated analytical measurements conducted under the same experimental conditions. Triplicate replicates applied to phytochemical and cytotoxicity assays, whereas extraction and fractionation procedures were conducted once using a representative bulk sample. Data were expressed as mean ± standard deviation (SD) from triplicate measurements. Confidence intervals (95% CI) were not calculated because the analysis focused on descriptive comparisons and concentration–response estimation based on technical replicates. Data were analyzed utilizing Tukey’s one-way analysis of variance (ANOVA) through Minitab^®^ 19 for Windows (Minitab, NSW, Australia). A notable difference was deemed significant at the *p* ≤ 0.05 level utilizing Tukey’s HSD test. The half-maximal inhibitory concentration (IC₅₀) and lethal concentration (LC₅₀) values were estimated from concentration–response curves by plotting extract concentration against percentage inhibition or mortality, respectively. Values were calculated using regression analysis to determine the concentration required to inhibit cell viability or induce 50% mortality.

## Results and Discussion

### Phytochemical Constituents

The phytochemical constituents of *H. durvillaei* were quantitatively analyzed using established colorimetric assays, as described in "Phytochemical Quantitative Analysis" section. Alkaloid, flavonoid, tannin, saponin, and steroid contents were determined using Dragendorff-thiourea, aluminum chloride, Folin–Denis, Liebermann-Burchard, and cholesterol-based colorimetric methods, respectively, following previously reported protocols [24–29].

**Fig. 1:**
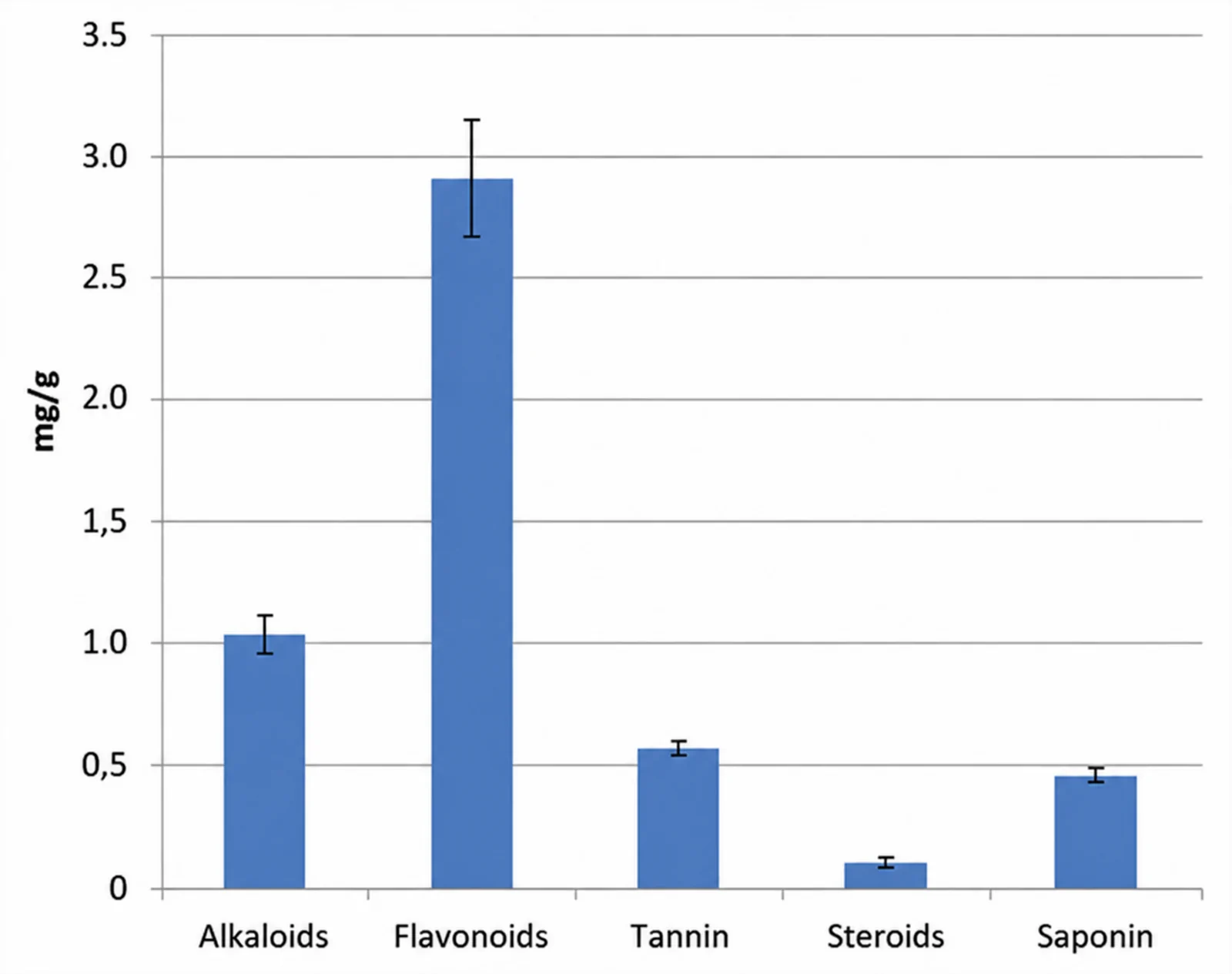
Phytochemical constituents identified in the methanolic extract of *H. durvillaei.* The extract comprises various compounds, including alkaloids, flavonoids, tannins, steroids, and saponins. Data are presented as mean ± standard deviation (*n* = 3)

It can be observed that nearly 50% of the 175 small molecules approved for cancer therapy are derived from natural compounds or their direct derivatives, which also serve as the foundation for many widely today used pharmaceuticals [43]. In the current investigation, alkaloids, flavonoids, tannins, saponins, triterpenoids, and phenolic compounds were identified in *H. durvillaei*. Figure 1 provides a summary of the phytochemical components found in *H. durvillaei*. The seaweed extract contains substantial amounts of flavonoids (2.91 ± 0.24 mg quercetin equivalents per gram of extract) and alkaloids (1.03 ± 0.08 mg/g extract). Additionally, *H. durvillaei* contains tannins (0.5683 ± 0.03 mg tannic acid equivalents per gram of extract), steroids (0.1057 ± 0.02 mg cholesterol equivalents per gram of extract), and saponins (0.4581 ± 0.03 mg per gram of extract). These compounds exhibit diverse biological activities and hold potential for use in the development of novel pharmaceuticals or dietary supplements.

Seaweed extract is known to contain a wide range of compounds, including tannins, saponins, flavonoids, terpenoids, triterpenoids, cardiac glycosides, steroids, and phenolic compounds [44, 45]. Previously, research has shown that extracts from *H. durvillaei* exhibit significant cytotoxic effects against various cancer cell lines, including human cervical adenocarcinoma. These effects are primarily attributed to the induction of apoptosis and modulation of cell proliferation pathways [16, 21]. For example, studies have demonstrated that hexane extracts of *H. durvillaei* can trigger apoptotic cell death in glioblastoma cells, suggesting a potential similar effect on cervical cancer cells [16]. The presence of bioactive alkaloids and phenolic compounds in *H. durvillaei* has been associated with its ability to inhibit tumor growth and induce cancer cell death, highlighting its promise as a candidate for further investigation in cancer therapy [18, 46].

Seaweeds are abundant in polyphenolic compounds, including phlorotannins, bromophenols, flavonoids, phenolic acids, terpenoids, and mycosporine-like amino acids [8]. Flavonoids, tannins, phenolics, sterols, and terpenoids function as a defensive mechanism against numerous viral diseases [47]. Furthermore, various macroalgae exhibit cytotoxic characteristics, and certain specialists propose the consumption of algae as a potential cancer prevention strategy [48]. Terpenoids comprise the predominant class of compounds exhibiting cytotoxicity against tumor cell lines, while bromophenols, phlorotannins, and alkaloids constitute a comparatively minor fraction [49]. Dimeric sesquiterpene and halogenated monoterpene demonstrate IC_50_ values against six distinct cancer cell lines (lung, cervical, gastric, hepatic, ileal, and colonic) ranging from 1 to 5 µM. Terpenoid extracts from *Cystoseira abies-marina* have demonstrated the capacity to inhibit HeLa cell proliferation [43].

Extracts of phlorotannins from *Laminaria japonica*, a species of brown algae, have exhibited anti-proliferative properties [50]. Furthermore, numerous saponin compounds have been recognized as significantly cytotoxic [51]. A brassinosteroid-related metabolite, isolated from *Cystoseira myrica*, demonstrated antiproliferative activity against HepG2 and HCT116 cancer cells [52]. Rhodophyta, or red seaweeds, are abundant in indole alkaloids, whereas Chlorophyta, or green seaweeds, predominantly possess halogenated alkaloids, particularly those with bromine and chloride [53]. Alkaloids derived from seaweeds encompass indoles and their analogs, quinone, 2-phenylethylamine, and 2,7-naphthyridine derivatives. Indole and 2-phenylethylamine are the predominant categories of alkaloids [54].

Research indicates that seaweed demonstrates considerable variability in its phytochemical composition. The red algae *Pterocladia capillacea* contains 0.93 mg/g of phenol, 0.46 mg/g of flavonoid, 20.68 mg/g of tannin, and 0.092 mg/g of carotenoid. The red seaweed *Corallina mediterranea* contained 0.37 mg/g of phenol, 0.12 mg/g of flavonoid, 13.33 mg/g of tannin, and 0.019 mg/g of carotenoid [55]. A separate study [56] reported that the red seaweed *Kappaphycus alvarezii* contained a phenolic content of 49.04 ± 6.05 mg GAE/100 g sample and a flavonoid content of 15.54 ± 1.68 mg CE/100 g sample, with a DPPH IC_50_ value of 18.2 ± 1.11. Flavonoids, a category of naturally occurring phenolic compounds with varied chemical structures, exhibit numerous biological activities, including antioxidant and free radical scavenging properties, which may serve as therapeutic agents for various diseases [55].

### BSLT Cytotoxic Activity

**Fig. 2:**
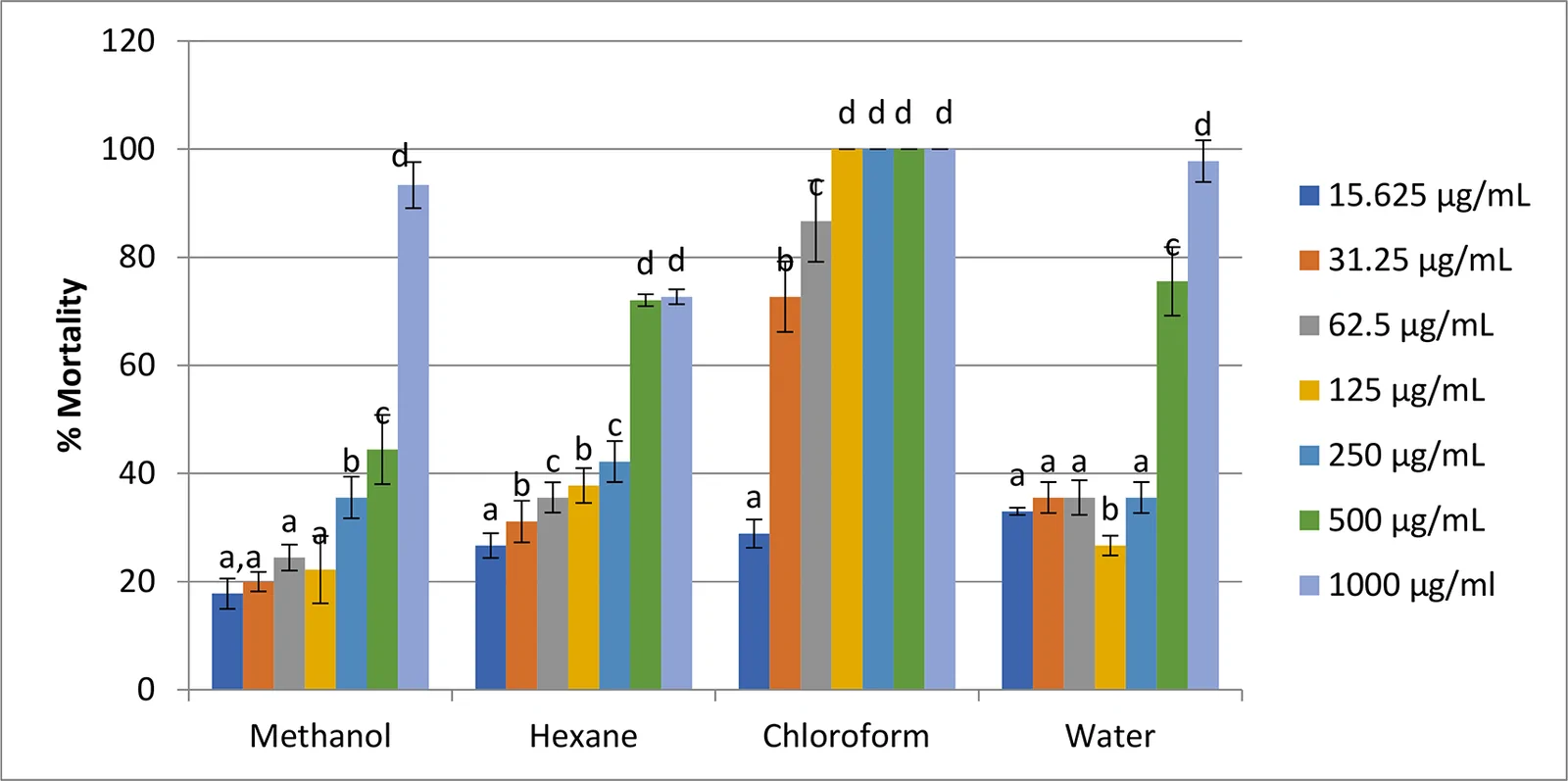
The mortality rate of nauplii following incubation in the methanolic extract, n-hexane fractions, chloroform fractions, and aqueous fractions (spanning 15.625-1,000 µg/mL) of *H. durvillaei.* Data are presented as mean ± standard deviation (*n* = 3). The superscript letters (a–d) denote significant differences (*p* < 0.05) among the various concentrations of the extract and its fractions

The Brine Shrimp Lethality Test (BSLT) serves as an effective method for preliminary toxicity assessment of marine products and for identifying pharmacological activity in plant extracts. BSLT’s capacity to identify various pharmacological activities demonstrates its significant association with the detection of antitumor compounds in both terrestrial plant extracts and marine plant extracts [30, 57]. BSLT testing demonstrated that the bioactive compound of *H. durvillaei* exhibits cytotoxic properties, as illustrated in Fig. 2. At concentrations of 500 ppm (equivalent to µg/mL), the n-hexane, chloroform, and water fractions exhibited a mortality rate surpassing 75%, signifying considerable cytotoxicity. The chloroform fraction demonstrated the highest mortality rate exceeding 75% at concentrations ranging from 31.25 to 1000 µg/mL, signifying substantial inhibition. The BSLT test results demonstrated non-cytotoxicity when mortality was below 50%, mild cytotoxicity when mortality ranged from 50% to 75%, and high cytotoxicity when mortality surpassed 75% [58].

The determination of the LC_50_ value employs the method of “The normal population assumption” or probit analysis. This method necessitated the administration of doses evaluated as logarithmic equivalent comparisons. According to the probit analysis, a logarithmic relationship curve was constructed for the test sample concentration and the probit value in Fig. 2. The methanolic extract, n-hexane, chloroform, and aqueous fractions of *H. durvillaei* demonstrated toxic activity, with LC_50_ values between 33.834 and 431 µg/mL (Table 1). The chloroform fraction exhibited the greatest activity, whereas the methanolic extract displayed the least. The methanolic extract and solvent fractions of *H. durvillaei* exhibited varying levels of toxicity based on their LC₅₀ values. According to toxicity classification criteria, compounds with LC₅₀ values < 30 ppm are categorized as highly toxic, those ranging from 30 to 1,000 ppm as toxic, and those > 1,000 ppm as non-toxic. In the present study, the methanolic extract and tested fractions were evaluated relative to these toxicity thresholds, indicating the presence of bioactive constituents with potential cytotoxic properties. Notably, lower LC₅₀ values indicate greater toxicity, which has been associated with increased potential for anticancer activity [58].

**Table 1:** The results of a probit analysis that expresses the LC_50_ value for brine shrimp toxicity

Samples	LC_50_ (µg/mL)
Methanolic extract	431.712
n-Hexane fraction	343.661
Chloroform fraction	33.834
Water fraction	242.280
Potassium dichromate (positive control)	11.350

The BSLT assay is widely used as a preliminary screening method to evaluate the cytotoxic potential of natural products prior to more complex pharmacological testing. Previous studies have reported correlations between BSLT toxicity and cytotoxic activity against several human cancer cell lines, including lung carcinoma (A-549) and colon carcinoma (HT-29), as well as certain solid tumors, supporting its utility as an early indicator of anticancer potential [30, 58, 59]. In the present study, the observed toxicity of *H. durvillaei* extracts in the BSLT assay suggests the presence of bioactive compounds that may contribute to cytotoxic effects against cancer cells, thereby supporting further evaluation using the HeLa cervical cancer cell line. The assay was performed using *Artemia salina* nauplii at an appropriate developmental stage, where sensitivity to test compounds is reported to be highest, thereby improving the reliability of toxicity assessment [30]. Nevertheless, more sensitive assays for anticancer activity, particularly those employing human cancer cells, remain essential. The findings of this study suggest that *H. durvillaei* may possess potential cytotoxic compounds. These compounds may be investigated further as innovative agents for cancer chemoprevention and adjunctive chemotherapy.

### Cytotoxic Activity to Cervical Cancer Cell Line

Our findings indicated that *H. durvillaei* exhibits considerable cytotoxic activity. The cytotoxic activity of *H. durvillaei* methanolic extract was not significant at concentrations ranging from 0.125 to 2 mg/mL. The outcome is illustrated in Table 2, which also shows the inhibition of cells in other fractions with an inhibition level ranging from 70.2 ± 10.34% to 93 ± 5.76%. Following fractionation, there was a notable increase in cytotoxic activity against cervical cancer cells. This discovery aligns with previous studies demonstrating that ethyl acetate extracts of *H. durvillaei* suppressed the proliferation of various cancer cell lines [60]. Furthermore, 3-(Hydroxyacetyl) indole and Indole-3-carboxylic acid derived from *H. durvillaei* have been documented to exhibit anti-lung cancer properties [61]. The anticancer efficacy of crude extracts from *H. durvellaei* was examined against four cancer cell lines: A549, HT-29, PC-3, and AGS. The findings demonstrated that the administration of *H. durvillaei* extracts inhibited the proliferation of AGS and HT-29 cell lines by 27.17% and 1.47%, respectively [33].

**Table 2:** Cytotoxic activity of the extracts and fractions of *H. durvillaei* against HeLa cell lines

Samples	Percentage of the inhibition (mg/mL)		
	0.125	0.5	2
Methanolic	**-**	**-**	**-**
Ethyl acetate	89.1 ± 7.22	83.9 ± 5.42	78.2 ± 9.24
n-Hexane	93.0 ± 5.76	90.5 ± 8.24	84.8 ± 7.67
Chloroform	70.2 ± 10.34	83.4 ± 9.25	78.2 ± 8.42
Water	78.2 ± 6.72	76.3 ± 5.24	89.2 ± 9.25

Most malignant cells share common characteristics that require examination to advance the development of anticancer therapies and treatment strategies [62]. Marine natural products function as inhibitors of cancer proliferation by obstructing mechanisms including anti-tubulin activity, apoptosis induction, and autophagy, as well as exhibiting anti-angiogenic, anti-migration, anti-invasion, and anti-metastatic properties, alongside proliferation inhibition and modulation of mitogen-activated protein kinases (MAPKs) [12, 63–65]. Table 3 illustrates the inhibition mechanisms of different varieties of seaweed.

**Table 3:** Cytotoxic mechanism of several types of seaweed against cervical cancer cells

Seaweed species/compound	Cytotoxic activity	Mechanism	References
*S. oligocystum*	IC_50_ 2.6375 ± 0.16 mg/mL	Anti-proliferation	This Study
*H. durvillaei*	IC_50_ 7.615 ± 0.32 mg/mL^− 1^	Anti-proliferation	This Study
		Induction of cell-cycle arrest (G2/M), apoptosis	Settacomkul et al., [91]
		Lipid components (e.g., oleic acid) may modulate pro-apoptotic signaling (BAD/Bax) and phosphatase targets (VHR)	Chantree et al., [92]
*Laurencia tristicha*	IC_50_ 15.5 µg/mL	Anti-tubulin	Sun et al., [93]
		Apoptosis	
*G. corticata*	IC_50_ 244.7 µg/mL	Anti-proliferation	Ashwini &amp; Shantaram, [94]
*S. filipendula* (Fukosantin)	4.717% (100 µg/mL)	Apoptosis	Zailanie et al., [69]
*Sargassum* sp. (Fucoidan)	IC_50_ 24 µg/mL	Apoptosis	Rafi &amp; Veerichetty, [95]
*S. ilicifolium*	IC_50_ 60 ± 0.2 µg/mL	Apoptosis	Namvar et al., [96]
*Dictyota cilliolata*	50% (0.2 mg/mL)	Apoptosis	Gomes et al., [23]
*Udotea flabellum*	22.5 µg/mL	Anti- proliferation	Majumder et al., [97]
*Enteromorpha intestinales*	309.048 ± 3.083 µg/mL	Anti- proliferation	Majumder et al., [97]
*Padina pavonia*	86.45 µg/mL	Anti- proliferation	Stanojkovic et al., [98]

HPV16 engages with the L1 major capsid protein to attach to heparin sulfate proteoglycans (HSPGs) located on the epithelial cell surface or the basement membrane [66]. Depending on the HPV type, the virus may infiltrate the host cell via clathrin or caveolin-mediated endocytosis [67]. Consequently, these proteins serve as targets for drug development aimed at inhibiting viral entry into cells and, subsequently, the onset of cancer [68]. Bioactive compounds derived from seaweed exhibit anticancer properties. Fucosantin, extracted from *S. filipendula*, inhibited HeLa cervical cancer cells by inducing mitochondrial apoptosis in the cytosol, while reducing the anti-apoptotic factor Bcl-2 and elevating the apoptosis-inducing factor Bax. Zailanie et al., [69]. A study indicates that certain sulfated polysaccharides derived from brown algae possess antiviral and antitumor properties. Several of these have been formulated into innovative antiviral agents [70]. Fucan is a category of sulfated polysaccharides containing L-fucose, located in brown seaweed, with structural variations observed among species and, in certain instances, within different sections of the seaweed [71].

Alginates, ulvans, and their sulfated derivatives, along with dextran sulfate and chitosan sulfate, are marine heparinoid polysaccharides exhibiting structural resemblance to heparin and biological characteristics akin to glycosaminoglycans [72]. Sulfated polysaccharides, including heparin, cellulose sulfate, and dextran sulfate, have been shown to inhibit papillomavirus infection [73]. The primary attachment of the virion to host cells is believed to be facilitated by interactions between the virion and the cell surface glycosaminoglycan (GAG) known as heparan sulfate, particularly for various viruses, including papillomaviruses [74].

The cytotoxic activity observed in *H. durvillaei* extracts may be partly associated with bioactive fatty acids such as oleic acid, which was identified as one of the predominant compounds in the GC–MS profile. Previous studies have reported that oleic acid can exert antiproliferative effects through modulation of cell-cycle progression, including G2/M phase arrest, and induction of apoptosis-related signaling pathways in cancer cells [75, 76]. These findings provide a plausible mechanistic basis linking the observed BSLT toxicity and HeLa cytotoxicity with the subsequent computational analysis of oleic acid as a representative bioactive compound.

### Molecular Docking

**Fig. 3:**
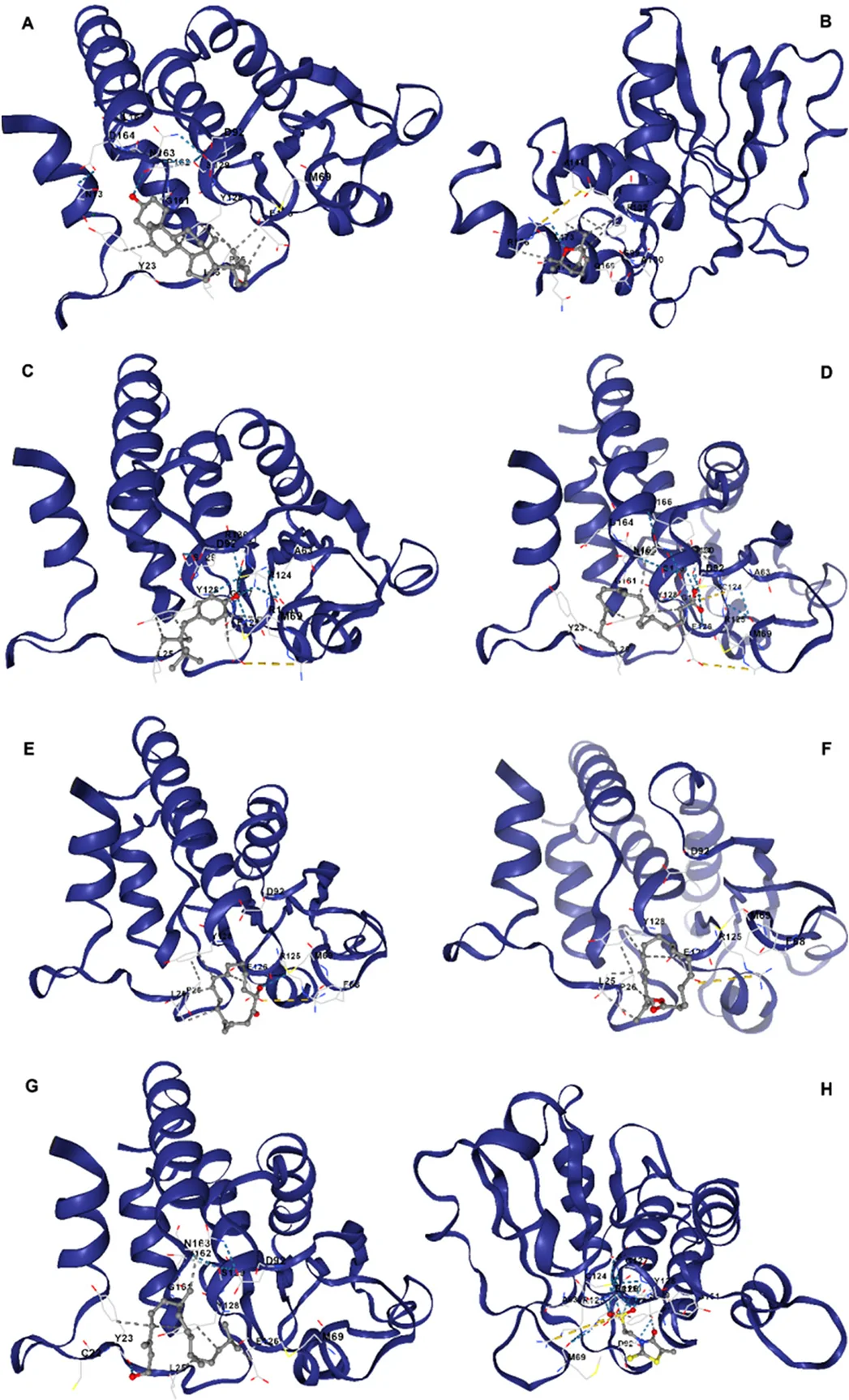
Binding poses of the compounds (**a**) 304, (**b**) 2758, (**c**) 41,234, (**d**) 445,639, (**e**) 543,400, (**f**) 557,450, (**g**) 5,366,244 and (**h**) cocrystal ligand STT in Vaccinia H1-related (VHR) active site obtained from AutoDock Vina molecular docking

Molecular docking is a computational modeling technique employed to anticipate the position and orientation of a complex formed by two molecules [77]. The results of the molecular docking analysis in this study are shown in Table 4, while Fig. 3 illustrates the binding conformations of the compounds examined at the active site of the Vaccinia H1-related phosphatase (VHR).

Table 4 solely presents the compounds with the highest binding affinity, reflecting the lowest binding-free energy (BFE) among those docked to the VHR. Protein tyrosine phosphatases (PTPs) have recently been associated with numerous human diseases, including cancer, and are proposed as potential pharmacological targets.

Vaccinia H1-related (VHR) phosphatase is a diminutive member of the dual-specificity phosphatase subclass, comprising merely 185 amino acids (Mr 21 kDa) and lacking a discernible targeting domain or docking site. VHR is a protein associated with cervical cancer, and its inhibition can diminish the proliferation of cervical cancer cells [78].

However, these computational findings should be interpreted with caution. The present docking results do not demonstrate that VHR is the actual intracellular target responsible for the observed antiproliferative effect in HeLa cells. Rather, they provide a preliminary hypothesis that may guide future mechanistic studies involving target validation, enzyme assays, and pathway-level confirmation.

**Table 4:** The outcomes of molecular docking simulations aimed at forecasting the orientation and position of a complex that might be created when the compounds from GC-MS interact with the VHR

Compounds	PubChem ID	Vina Score (Kcal/mol)	Cavity volume (Å^3^)	Center (x, y, z)	Docking size (x, y, z)	Contact Residues
Cholest-5-En-3-ol	304	-6.5	69	3, 7, -6	25, 25, 25	Asn13, Tyr23, Leu25, Pro26, **Asp92**, Glu126, Tyr128, Gly161, Pro162, Asn163, Asp164, Leu167
Eucalyptol	2758	-4.0	69	3, 7, -6	16, 16, 16	Leu25, Pro26, Met69, **Asp92**, Arg125, Glu126, Tyr128, Ser129, **Arg130**, Gly161
Phenol, 4-(2,2,3,3-Tetramethylbutyl)	41,234	-5.0	69	3, 7, -6	19, 19, 19	Leu25, Met69, **Asp92**, Cys124, Arg125, Glu126, Gly127, Tyr128, Ser129, **Arg130**
Oleic acid	445,639	-4.8	69	3, 7, -6	23, 23, 23	Tyr23, Leu25, Met69, **Asp92**, Cys124, Arg125, Glu126, Gly127, Tyr128, Ser129, **Arg130**, Gly161, Pro162
2-Hydroxycyclopentadecanone	543,400	-4.3	69	3, 7, -6	19, 19, 19	Leu25, Pro26, Met69, **Asp92**, Arg125, Glu126, Tyr128
1-Oxacyclopentadecan-2-one, 15-isopropenyl	557,450	-5.3	69	3, 7, -6	20, 20, 20	Leu25, Pro26, Met69, Asp92, Glu126, Tyr128
Phytol	5,366,244	-4.1	69	3, 7, -6	22, 22, 22	Cys22, Tyr23, Leu25, Met69, **Asp92**, Glu126, Tyr128, Ser129, Gly161, Pro162, Asn163
2-(5-methyl-4-oxo-2-sulfanylidene-1,3-thiazolidin-3-yl)ethanesulfonic acid	STT	-5.2	69	3, 7, -6	19, 19, 19	Met69, **Asp92**, Cys124, Arg125, Glu126, Gly127, Tyr128, Ser129, **Arg130**, Gly161

The compounds demonstrating the highest binding affinity, signifying the lowest binding-free energy (BFE) among those docked to the VHR. Protein tyrosine phosphatases (PTPs) have recently been associated with numerous human diseases, including cancer, and are proposed as potential pharmacological targets. Vaccinia H1-related (VHR) phosphatase is a diminutive member of the dual-specificity phosphatase subclass, comprising merely 185 amino acids (M_r_ 21 kDa) and lacking a discernible targeting domain or docking site. VHR is a protein associated with cervical cancer, and its inhibition can diminish the proliferation of cervical cancer cells [78].

This study utilized the small molecule inhibitor of VHR, 2-(5-methyl-4-oxo-2-sulfanylidene-1,3-thiazolidine-3-yl)ethanesulfonic acid (PubChem ID: STT), as a comparative reference. The compound STT exhibited a binding-free energy (BFE) of -5.2 kcal. Prior studies have demonstrated that VHR possesses two notable pockets: an active site cleft formed by Asp92, Arg130, and Ser124, and an additional basic pocket, Arg158 [79]. The compounds eucalyptol, phenol, 4-(2,2,3,3-tetramethylbutyl), oleic acid, and STT engage with the residues Asp92 and Arg130 situated at the active site of VHR.

### Parmacokinetic Properties

The ADMET profile of the ligand oleic acid (Table 5) exhibited a high human intestinal absorption (HIA) value of 98.436935, an in vivo blood-brain barrier penetration of 7.48966, in vitro P-glycoprotein inhibition, in vitro plasma protein binding of 100%, skin sensitivity of 0.539417, and indicated that oleic acid is carcinogenic to rats and mice.Ligands derived from oleic acid in *H. durvillaei* exhibited high HIA absorption, making them suitable for the design of new oral medications. The influence of the blood-brain barrier (BBB) on the central nervous system is perilous if a drug acts solely at the peripheral level. Oleic acid exhibits favorable blood-brain barrier permeability. The P-glycoprotein substrate is crucial for the transport of a drug that affects absorption and distribution. The inhibition of cytochrome P450 enzymes, which are responsible for drug metabolism, may lead to elevated plasma levels and toxicity. The in silico calculations indicate that oleic acid is not a respective inhibitor of these cytochrome P450 enzymes. The Log Kp of -0.539417 A, B cm/s does not indicate skin sensitivity. A BiA score of 1 indicates that the compound is a promising candidate for therapeutic application [80]. Oleic acid exhibited BiA values approaching 1, indicating promising theoretical data for the development of novel pharmaceuticals. A drug-likeness score quantifies the theoretical resemblance of a compound to an established therapeutic agent. All ADMET parameter profiles of oleic acid exhibit biological activity that may have potential implications for cervical cancer in the future.

**Table 5:** Profile ADMET of Oleic Acid

No.	Biological Activity	Value
1.	AlogP98 (calculated logP by Ghose method)	6.835400
2.	A MolRef (calculated atomic molecular refractivity by Ghose method)	87.402200
3.	in vivo blood-brain barrier penetration/BBB (C.brain/C.blood)	7.48966
4.	Water solubility in buffer system (SK atomic types, mg/L)	153.761
5.	in vitro Caco^− 2^ cell permeability (nm/sec)	28.1906
6.	in vitro Cytochrome P450 2C19 inhibition Value	Inhibitor
7.	in vitro Cytochrome P450 2C9 inhibition	Inhibitor
8.	in vitro Cytochrome P450 2D6 inhibition	Inhibitor
9.	in vitro Cytochrome P450 2D6 substrate	Non
10.	in vitro Cytochrome P450 3A4 inhibition	Inhibitor
11.	in vitro Cytochrome P450 3A4 substrate	Non
12.	Human intestinal absorption (HIA, %)	98.436935
13.	in vitro MDCK cell permeability (nm/sec)	70.3359*
14.	in vitro P-glycoprotein (PGP) inhibition	Inhibitor
15.	in vitro plasma protein binding (%)	100.000000
16.	Water solubility in pure water (SK atomic types, mg/L)	0.916548
17.	in vitro skin permeability (logKp, cm/hour)	-0.539417
18.	Water solvation free energy (Ghose method)	-4.830000
19.	Acute algae toxicity	0.00222903
20.	Ames test	mutagen
21.	Carcinogenicity(Mouse)	positive
22.	Carcinogenicity(Rat)	positive
23.	Acute daphina toxicity	0.00637744
24.	in vitro hERG inhibition	low_risk
25.	Acute fish toxicity (medaka)	6.4643e-005
26.	Acute fish toxicity (minnow)	3.73882e-005
27.	in vitro Ames test result in TA100 strain (Metabolic activation by rat liver homogenate)	negative
28.	in vitro Ames test result in TA100 strain (No metabolic activation)	negative
29.	in vitro Ames test result in TA1535 strain (Metabolic activation by rat liver homogenate)	negative
30.	in vitro Ames test result in TA1535 strain (No metabolic activation)	negative

### Molecular Dynamics Simulations Study

**Fig. 4:**
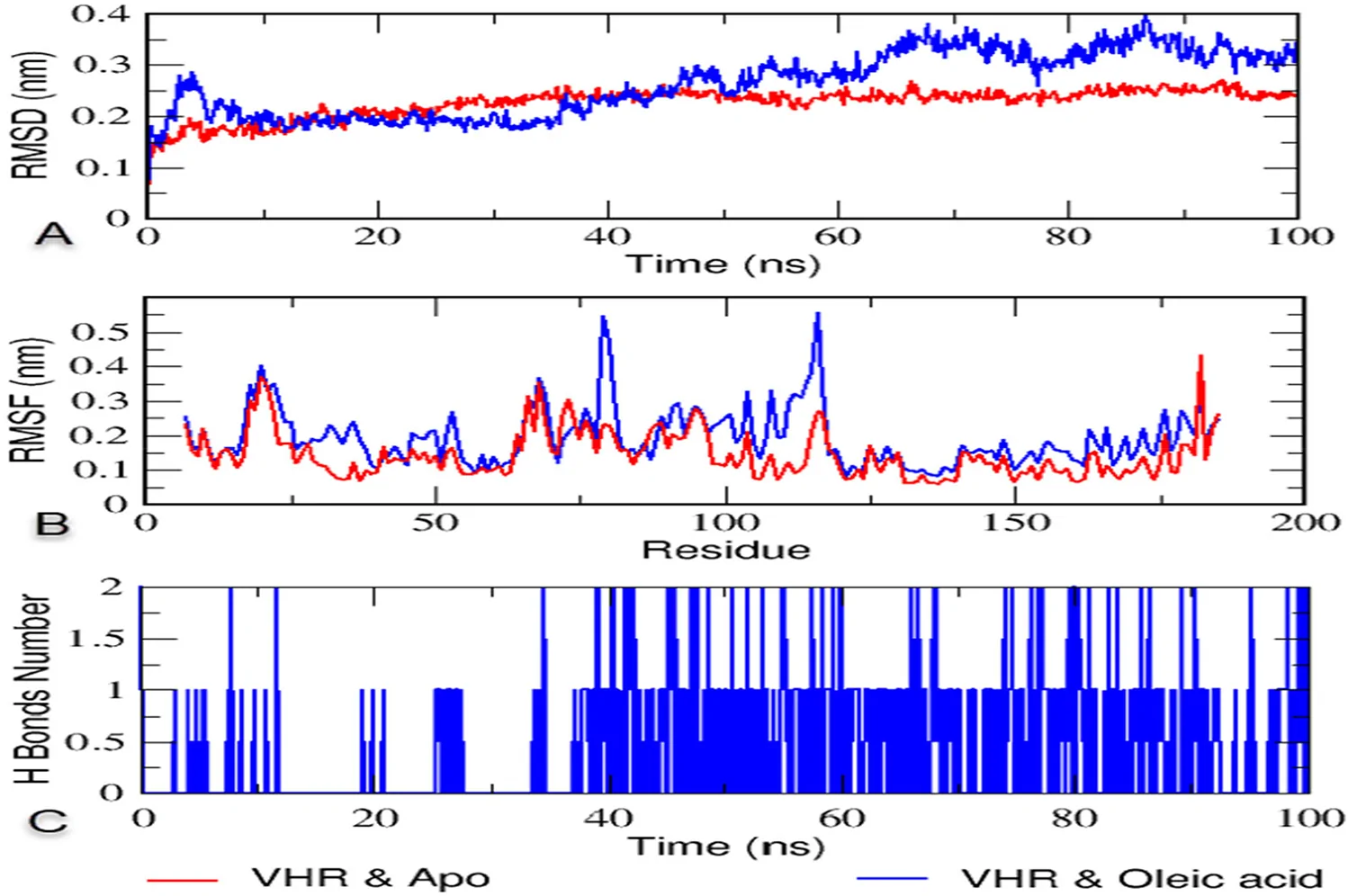
Molecular dynamics simulation: (**a**) RMSD; (**b**) RMSF; and (**c**) H-bonds analysis of VHR and oleic acid complex as well as VHR apoprotein

Based on molecular docking results and existing data, oleic acid demonstrates potential as an anticancer agent. Consequently, it was chosen for simulation to examine its dynamics. The objective of this simulation was to assess the stability of the compound. A 100-nanosecond molecular dynamics simulation of the oleic acid and VHR complex, along with the apoprotein, was conducted and illustrated in Fig. 4.

The stability of the complex was evaluated by measuring the root-mean-square deviation (RMSD) of the complex and contrasting it with that of the apoprotein. The RMSD quantifies the comparison between a protein’s initial and final structural conformations, with the deviations observed during simulation indicating the stability of the protein’s structure. Stable protein structures exhibit smaller deviations in the protein backbone compared to less stable ones [81].

The results demonstrate that both structures underwent a marginal rise in RMSD values during the simulation. The apo-VHR structure (red) exhibited reduced fluctuations relative to the oleic acid-VHR structure (blue). The RMSD value for apo-VHR fluctuated between 0.10 and 0.25 nm from 0 to 40 ns, subsequently stabilizing at approximately 0.25 nm for the duration of the 100 ns simulation. The RMSD value for the oleic acid-VHR complex varied between 0.15 and 0.40 nm during the first 40 ns, peaking at approximately 84 ns. The elevated RMSD value at the conclusion of the simulation, particularly noted in the oleic acid-VHR complex, signifies structural alterations that can be employed to evaluate the integrity of the structure. Nonetheless, the variations persisted beneath 0.4 nm. The elevated RMSD value in the oleic acid-VHR complex was ascribed to enhanced atomic mobility. The relative stability of apo-VHR and the oleic acid–VHR complex was interpreted based on the combined evaluation of RMSD, RMSF, radius of gyration, SASA, and trajectory convergence, rather than hydrogen bond interactions alone. Hydrogen bond analysis served as complementary evidence of ligand–protein interaction stability in the oleic acid-bound system. Reduced fluctuations in the RMSD value indicate diminished shifts and deviations in the protein structure of the complex [82, 83].

An analysis of root-mean-square fluctuations (RMSF) was conducted, indicating that both apo-VHR and oleic acid-VHR complexes displayed analogous fluctuations across the majority of their residues; however, specific residues exhibited elevated and diverse fluctuations relative to others. The oleic acid-VHR complex exhibited marginally greater fluctuation compared to the apoprotein-VHR complex, as evidenced by the RMSD values. The oleic acid-VHR complex demonstrated fluctuations at the 70th and 120th residues, while the apo-VHR structure exhibited more pronounced fluctuations towards the conclusion of the 100 ns simulation. The RMSF value illustrates the conformational alterations of the complexes, signifying the flexibility of their protein structures. The RMSF values are diminished for residues located near the protein’s core and elevated for loop regions and surface residues [84]. Reduced fluctuation values indicate a more compact and stable conformation, characterized by diminished movement and a shorter distance between residues [83, 85]. Despite the minor differences between the two complexes, it can be concluded that the apo-VHR structure exhibited a more stable RMSF than the oleic acid-VHR complex. This may result from the unbound state of apo-VHR, in contrast to the bound oleic acid-VHR complex, which may have induced specific structural alterations, resulting in a degree of mobility in certain residues of the complex, especially those implicated in the binding interaction between oleic acid and VHR.

The folding and structure of proteins, as well as the recognition of molecules, are heavily dependent on hydrogen bonds (H-bonds). These bonds play a critical role in the formation of the secondary structure of proteins, which includes alpha helices and beta strands [86, 87]. Furthermore, the presence and quantity of H-bonds and polar surfaces have an impact on protein thermostability [88]. During the simulation process, it is possible for the number of H-bonds to fluctuate due to changes in atomic interactions. As a result, mostly one H-bond and occasionally two H-bonds were observed to form.

The conformation and architecture of proteins, along with molecular recognition, are significantly reliant on hydrogen bonds (H-bonds). These bonds are essential for the development of the secondary structure of proteins, comprising alpha helices and beta strands [86, 87]. The existence and amount of hydrogen bonds and polar surfaces influence protein thermostability [88]. Throughout the simulation process, the quantity of hydrogen bonds may vary due to alterations in atomic interactions. Consequently, primarily one hydrogen bond and intermittently two hydrogen bonds were observed to form.

Previous studies have demonstrated that *H. durvillaei* possesses anti-inflammatory, antioxidant, antimicrobial, anticancer, cardiovascular, neuroprotective, renal, and hepatic activities, which have been attributed to bioactive compounds such as eucalyptol, oleic acid, dodecanal, and pentadecane [33]. Moreover, oleic acid has been recognized as an anticancer agent that triggers apoptosis via a caspase-3-independent pathway, which entails the dephosphorylation of BAD, a constituent of the BCL2 family of proapoptotic proteins [89]. Furthermore, oleic acid has been shown to induce apoptosis and autophagy in tongue squamous cell carcinomas [90]. Mitogen-activated protein kinases (MAPKs) are a family of proteins that specifically phosphorylate the amino acids serine, threonine, and tyrosine, and are closely linked to numerous cellular functions in cancer, including cell differentiation, proliferation, growth, cell cycle regulation, apoptosis, migration, invasion, metastasis, energy metabolism, and angiogenesis [62].

### Conclusions

The findings demonstrate that *Halymenia durvillaei* exhibits measurable cytotoxic activity, with the n-hexane fraction showing the strongest inhibition of HeLa cell proliferation. Consistent toxicity patterns in the brine shrimp lethality assay further confirm the bioactivity of the n-hexane, chloroform, and water fractions. Phytochemical screening and in-silico analyses identified oleic acid as a major constituent with a favorable binding affinity toward targets associated with cervical cancer cell survival, supporting its potential pharmacological relevance. Collectively, these results indicate that *H. durvillaei* represents a promising source of bioactive compounds for anticancer drug discovery, triggering further mechanistic and compound-level validation through advanced chemical and cellular assays.

Despite the promising findings, the current study however has certain limitations. The molecular docking and dynamics simulations, while insightful, are in silico (computational) models and require in vitro (cell culture) and in vivo (animal) validation to confirm the predicted binding and efficacy of compounds like oleic acid. Also, the cytotoxic activity of the methanolic extract against HeLa cells was not significant at the tested concentrations (0.125 to 2 mg/mL), though its fractions proved active, suggesting the need for more efficient extraction/fractionation methods or perhaps different testing ranges for the crude extract. Future work should focus on isolating and characterizing the specific bioactive compounds responsible for the cytotoxic activity in the most active fractions (n-hexane, chloroform, and water). Comprehensive mechanistic studies are essential to elucidate how these compounds, especially oleic acid, induce cell death (e.g., through apoptosis or cell cycle arrest) in cervical cancer cells beyond the initial VHR phosphatase inhibition hypothesis, and to confirm the predicted minimal adverse effects suggested by the BSLT. Finally, further ADMET and animal model studies are crucial to determine the safe and effective therapeutic window for developing a novel pharmaceutical agent from *H. durvillaei* extracts.

## Data Availability

The data that support the findings of this study are available from the corresponding author upon reasonable request.

## Abbreviations

ADMET: Absorption, Distribution, Metabolism, Excretion, and Toxicity
AGS: Gastric adenocarcinoma cell line
ANOVA: Analysis of Variance
ATCC CCL-2: American Type Culture Collection, Cell Line (HeLa)
BBB: Blood-Brain Barrier
BFE: Binding-Free Energy
BiA: Bioactivity
BSL: Brine Shrimp Lethality Test
BSLT: Brine Shrimp Lethality Test
Caco-2: Human colon carcinoma cell line
CO2: Carbon Dioxide
DMEM: Dulbecco’s Modified Eagle Medium
DMSO: Dimethyl Sulfoxide
DPPH: 2,2-diphenyl-1-picrylhydrazyl (used for IC50 value in antioxidant activity)
DSPs: Dual Specificity Protein Phosphatases
FBS: Fetal Bovine Serum
FCS: Fetal Calf Serum
GAG: Glycosaminoglycan
HeLa: Human cervical adenocarcinoma cell lines/cells
HIA: Human Intestinal Absorption
HIV: Human Immunodeficiency Virus
HPV: Human Papillomavirus
HSPGs: Heparin Sulfate Proteoglycans
HT-29: Colon carcinoma cell lines
IC50: Half-maximal Inhibitory Concentration
LMICs: Low- and middle-income countries
MAPKs: Mitogen-Activated Protein Kinases
MD: Molecular Dynamics
MDCK: Madin-Darby canine kidney cell line
ME: Methanolic Extract
MM: Medium containing 5% FCS (or serum-free medium)
MTT: 3-(4,5-dimethylthiazol-2-yl)-2,5-diphenyltetrazolium bromide
NaCl: Sodium Chloride
NVT/NPT: Canonical ensemble (constant number of particles, volume, and temperature)/Isothermal-isobaric ensemble (constant number of particles, pressure, and temperature)
PBS: Phosphate-Buffered Saline
PC-3: Prostate cancer cell line
PDB ID 3F81: Protein Data Bank ID 3F81 (identifier for VHR phosphatase)
PGP: P-glycoprotein
ppm: parts per million
PTPs: Protein Tyrosine Phosphatases
QCE: Quercetin Equivalent
RMSD: Root-Mean-Square Deviation
RMSF: Root-Mean-Square Fluctuations
STT: 2-(5-methyl-4-oxo-2-sulfanylidene-1,3-thiazolidin-3-yl)ethanesulfonic acid
TFC: Total Flavonoid Content
VHR: Vaccinia H1-Related phosphatase
µg/mL: Micrograms per milliliter
mg/mL: Milligrams per milliliter
mg/g: Milligrams per gram
nm: Nanometer
